# Ensemble species distribution modeling and multilocus phylogeography provide insight into the spatial genetic patterns and distribution dynamics of a keystone forest species, *Quercus glauca*

**DOI:** 10.1186/s12870-024-04830-1

**Published:** 2024-03-04

**Authors:** Ying Song, Gang-Biao Xu, Ke-Xin Long, Chun-Cheng Wang, Ran Chen, He Li, Xiao-Long Jiang, Min Deng

**Affiliations:** 1 College of Forestry, The Laboratory of Forestry Genetics, Central South University of Forestry and Technology, Changsha, 410004 Hunan China; 2 College of Landscape Architecture, Central South University of Forestry and Technology, Changsha, 410004 Hunan China; 3 School of Ecology and Environmental Sciences, Yunnan University, Kunming, Yunnan 650500 China

**Keywords:** Oak, Dispersal corridor, Spatial genetic pattern, Subtropical evergreen broad-leaved forest, Climate change

## Abstract

**Background:**

Forests are essential for maintaining species diversity, stabilizing local and global climate, and providing ecosystem services. Exploring the impact of paleogeographic events and climate change on the genetic structure and distribution dynamics of forest keystone species could help predict responses to future climate change. In this study, we combined an ensemble species distribution model (eSDM) and multilocus phylogeography to investigate the spatial genetic patterns and distribution change of *Quercus glauca* Thunb, a keystone of East Asian subtropical evergreen broad-leaved forest.

**Results:**

A total of 781 samples were collected from 77 populations, largely covering the natural distribution of *Q. glauca.* The eSDM showed that the suitable habitat experienced a significant expansion after the last glacial maximum (LGM) but will recede in the future under a general climate warming scenario. The distribution centroid will migrate toward the northeast as the climate warms. Using nuclear SSR data, two distinct lineages split between east and west were detected. Within-group genetic differentiation was higher in the West than in the East. Based on the identified 58 haplotypes, no clear phylogeographic structure was found. Populations in the Nanling Mountains, Wuyi Mountains, and the southwest region were found to have high genetic diversity.

**Conclusions:**

A significant negative correlation between habitat stability and heterozygosity might be explained by the mixing of different lineages in the expansion region after LGM and/or hybridization between *Q. glauca* and closely related species. The Nanling Mountains may be important for organisms as a dispersal corridor in the west-east direction and as a refugium during the glacial period. This study provided new insights into spatial genetic patterns and distribution dynamics of *Q. glauca*.

**Supplementary Information:**

The online version contains supplementary material available at 10.1186/s12870-024-04830-1.

## Background

The geological record contains many examples of divergences in species lineages and distribution changes caused by either climate fluctuations or geologic activity [1–3]. Recent climate change, meanwhile, has affected ecosystem function and biodiversity patterns by altering forest community structure and species distribution patterns [4, 5]. Going forward, all evidence suggests rapid climate change will remain a primary threat to species and ecosystems, and these effects will intensify as the pace of change accelerates [6–8]. Since keystone species have outsized roles in ecosystem formation and community distribution dynamics [9], exploring the effects of paleogeological events and climate change on the spatial genetic patterns in keystone species can not only reveal the ancient drivers of speciation but also help to evaluate the distribution dynamics of ecosystems in the context of future climate change [10, 11].

Subtropical East Asia has experienced complex tectonic activity and climate change effects in recent geological records. These include the uplift of the Himalayan-Tibetan Plateau from about 50 Ma [12, 13], the aridification of Central Asia from about 22 Ma [14], and the formation of Asian monsoons 8–9 Ma [15]. These events significantly impacted East Asia’s landforms, topography, and climate, which profoundly affected genealogical differentiation, genetic diversity, and distribution of organisms [16–18]. The diverse microhabitats in the complex topography regions will provide suitable habitats for species surviving during climate change [17, 19], thus serving as refugia for many relict taxa [20, 21].

The primary ecosystem of subtropical East Asia is an evergreen broadleaved forest (EBLF), which stretches between 24–32°N and 99–123°E. EBLFs in subtropical East Asia comprise 2,600 genera and ca.14,600 species of seed plants, of which > 50% are endemic [22]. Conservation of this biodiversity in EBLFs in subtropical East Asia is the subject of much attention from researchers, especially in the face of ongoing rapid climate change.

The genus *Quercus* (oaks), which includes ca. 450 species, is widely distributed in the Northern Hemisphere. Besides providing wood and food, oaks are keystone species in different habitats [23]. Oaks can undergo frequent introgression events and are adaptable to short- and long-term environmental change [24–27]. The *Quercus robur* genome confirmed the intergenerational transmission of adaptive variants, for instance [28]. Genomic and anatomic data analysis indicated that oaks might have different defense mechanisms against pathogens [29]. Chloroplast DNA (cp.DNA) markers from *Q. acutissima*, *Q. chenii*, and *Q. variabilis* revealed haplotype sharing within section *Cerris* in East Asian EBLFs that was associated with locally stable climates and complex landscapes [30]. Based on SSR and phenotypic data in two oak species indicated that asymmetric inter-specific selection pressures could contribute to the asymmetric trait divergence where species coexist [31]. Resequencing data indicated that the introgression between two widespread sympatric Asian oak species, *Q*. *acutissima* and *Q. variabili*s, confers environmental adaptation by altering the regulation of stress-related genes [32]. These factors justify using oaks as a model species for understanding the development of evolutionary patterns from ecological processes.

*Quercus glauca* Thunb, one of the most widespread species in the *Quercus* section *Cyclobalanopsis*, is a keystone species in subtropical evergreen forests. Population genetic patterns and local adaptation in the species have been investigated in previous studies. For example, genotyping six *Q. glauca* populations from East China using six enzymes found high genetic diversity but low genetic differentiation [33]. Based on three cp.DNA loci, southeastern Taiwan Island was suggested to be a potential glacial refugium for *Q. glauca* [34]. Genotyping 409 individuals from 42 populations from China mainland and Japan using three cp.DNA loci, it was found that genetic differentiation of *Q. glauca* began in the Miocene and it may have experienced expansion after the Last Glacial Maximum (LGM; approximately 22,000 years ago) [35]. Although the samples in Xu et al. [35] roughly cover the current *Q. glauca* distribution, they lacked representation from Taiwan Island. Furthermore, cp.DNA can only reveal seed gene flow but without information about pollen gene flow. Different markers can reveal the evolutionary history of species more objectively [36, 37]. Finally, compared with the single model (MaxEnt) previously used to predict the species range of *Q. glauca* [35], the ensemble species distribution model (eSDM) can integrate multiple models and improve prediction accuracy [38, 39].

In this study, we combined nuclear microsatellites (*n*SSR), cp.DNA markers, and eSDM to investigate the spatial genetic patterns and distribution dynamics of *Q. glauca*. A total of 781 individuals from 77 populations were sampled. The samples were genotyped using seven *n*SSR and three cp.DNA loci. The eSDM was used to estimate the distribution dynamics of *Q. glauca* under predicted past and future climate change. Our goals were to (i) reveal spatial genetic patterns of *Q. glauca*, (ii) estimate the effect of environmental factors on genetic diversity, and (iii) infer potential dispersal corridors. Our study provides guidelines for preserving *Q. glauca* germplasm resources and managing the biodiversity of East Asia subtropical EBLFs in the context of future climate change.

## Results

### Ecological niche modeling

Based on the filtered 238 distribution points and nine environmental variables (Fig. S1), nine niche models were used to predict the potential distribution of *Q. glauca* (Fig. S2). Among the nine models, RF, GLM and GBM have higher mean TSS and AUC values, and SRE has the lowest TSS and AUC values (Fig. S2 and Table S1). After excluding nine models from SRE, one model from CTA, and one model from MaxEnt with low evaluation (TSS < 0.6 and AUC < 0.8), the remaining 79 models were used to construct the ensemble model based on the weighted TSS (Table S1). The average TSS and AUC values for the remaining 79 models were 0.90 and 0.99, respectively. The predicted potential distribution of *Q. glauca* has an extensive suitable range in East Asia (Fig. S3). During the LGM period, the potential suitable distribution of *Q. glauca* was mainly in southern China, the southern slope of the Himalayas, the Korean Peninsula, and the East China Sea continental shelf. In the present period, southern China, Taiwan Island, and southwestern Japan show high suitability. In the future, due to climate change, southeast China and Japan will show high distribution probabilities, but the overall distribution and high suitability areas are predicted to diminish.

**Fig. 1:**
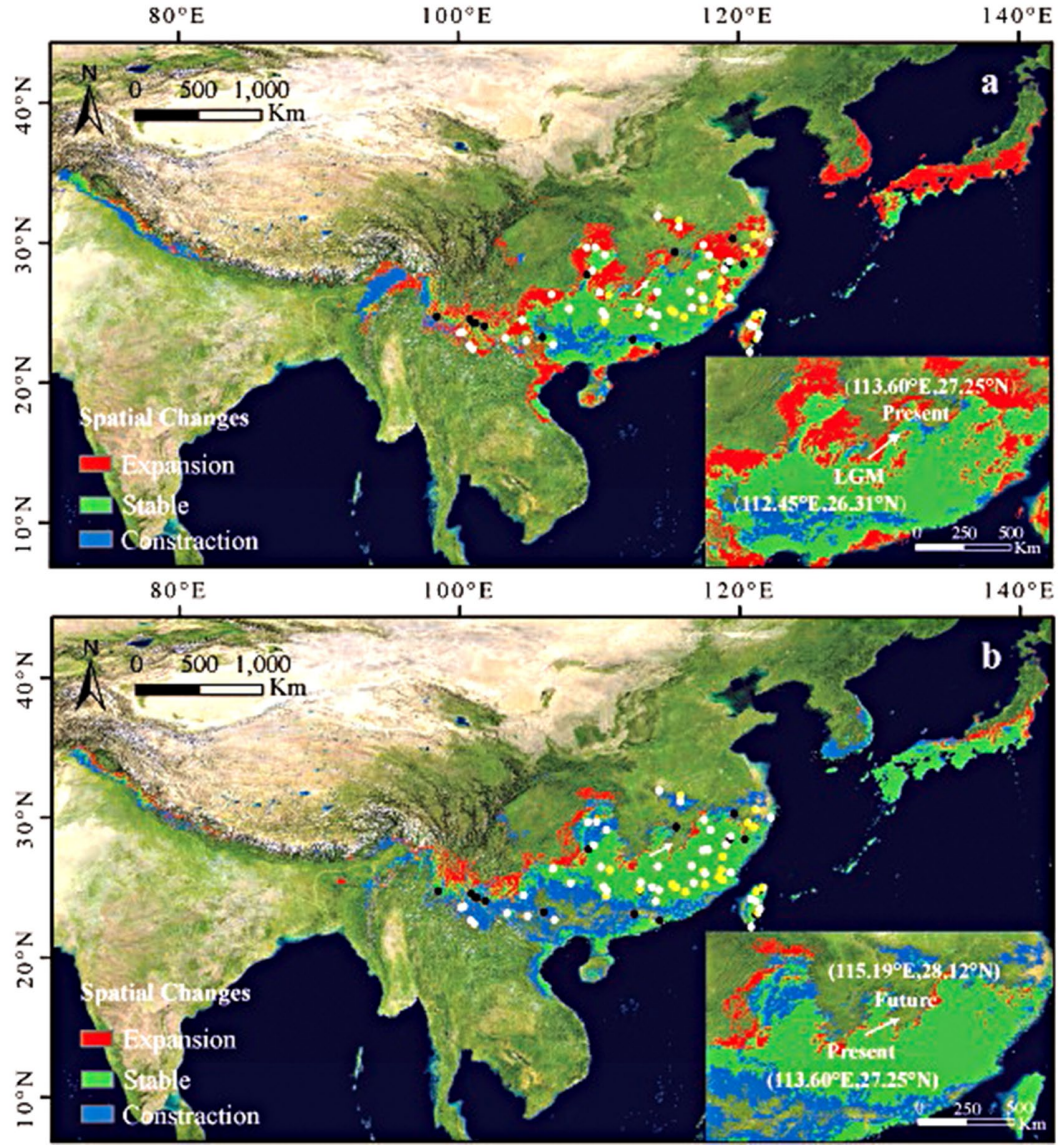
The distribution dynamic of *Quercus glauca* under climate change is based on the ensemble model and 238 distribution points. **a** the LGM (CCSM4) to present period (1970-2000s); **b** present to future period (Mean of four BCC scenarios, SSP126, SSP245, SSP370, and SSP585, in the 2081-2100s). The blue, green, and red regions represent the areas of loss, stablility and gain in response to climate change. The yellow, black, and white dots represent populations for *n*SSR analysis only (20 populations), for cp.DNA analysis only (17 populations), and for both *n*SSR and cp.DNA analysis (40 populations), respectively. The bottom right of each figure represents the change in the centroid of the species distribution in response to climate change

From the LGM to the present period, the suitable habitat of *Q. glauca* has expanded. The overall suitable habitat increased by 41%, with an area of 78.36 × 10^4^ km^2^ mainly concentrated in the northern portion of their distribution after a northeastern expansion (Fig. 1). In the future, the area will shrink by an estimated 33%, leaving habitat mainly concentrated in southern Korea and southwest China. According to the migration routes through the centroids, the shrinking occurs towards the northeast, similar to the expansion after the LGM.

### Genetic diversity and structure based on microsatellites

**Fig. 2:**
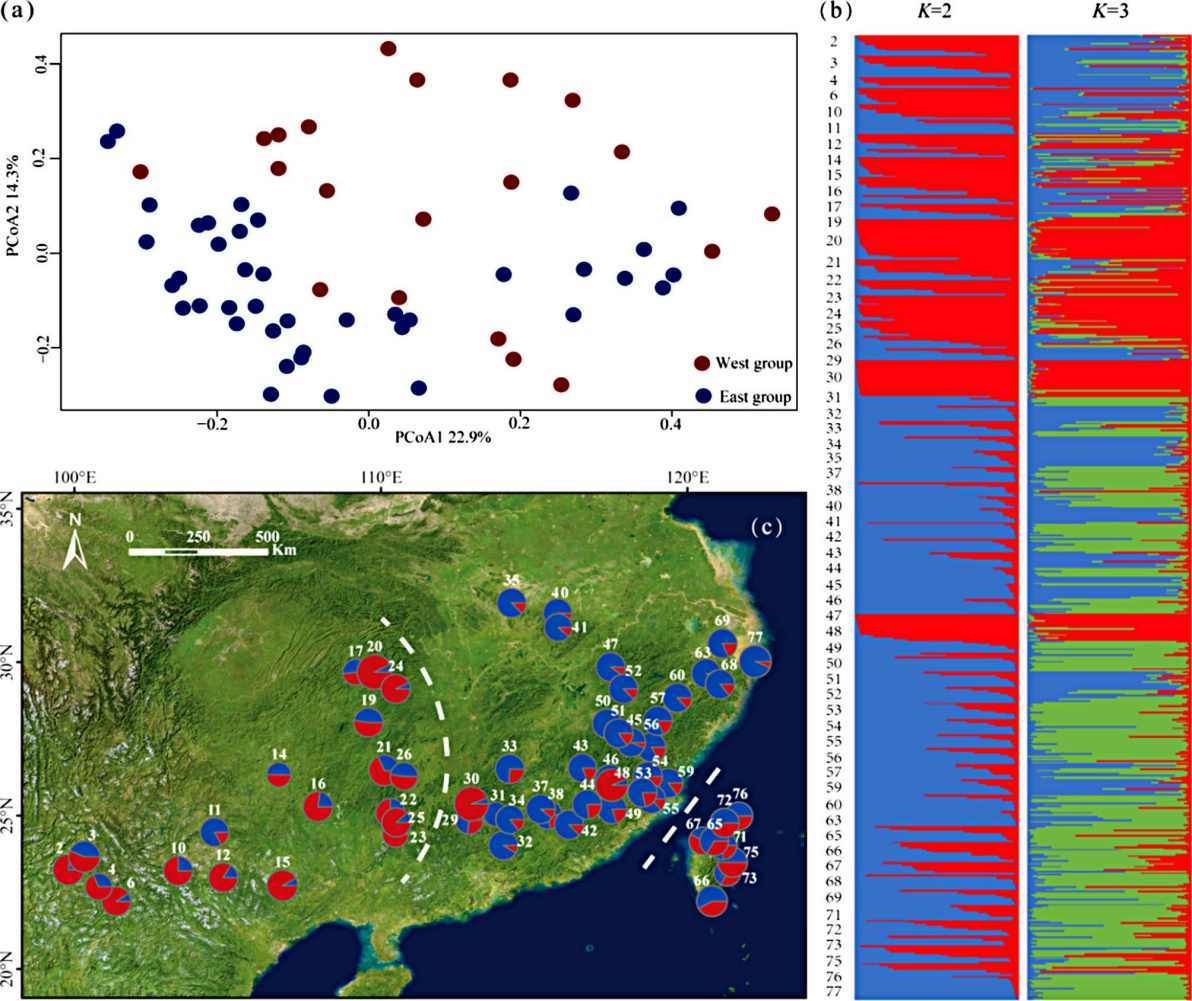
Structure analysis of 60 *Quercus glauca* populations. (**a**) Principal coordinates analysis (PCoA) of the 60 populations of the *Quercus glauca* based on genetic distance using *n*SSR data. (**b**) Bayesian clustering plots for 60 populations of *Quercus glauca* based 7 *n*SSR loci, *K* = 2 and *K* = 3 was presented (**c**) Geographical distribution of the genetic clusters and genetic cluster composition in each population. The color of the pie chart represents different groups, and the white dotted line represents the geographical obstacles based on barrier analysis

A total of 620 individuals from 60 populations were genotyped by seven *n*SSR primers (Table 1). The genetic diversity indices *H*_o_, *H*_e_, and *A*_r_ were 0.38–0.79 (mean: 0.57), 0.43–0.77 (mean: 0.60), and 2.88–5.97 (mean: 3.74), respectively (Table 1). The highest *H*_o_ and *H*_e_ were found in populations 76 and 30, respectively, and the lowest for both statistics was found in population 17. The *A*_r_ value of population 26 was the highest, and the lowest was found in population 32. The Pr(*X*|*K*) and Δ*K* support the presence of two major clusters of *Q. glauca* populations (Fig. S4). This result clustered 20 populations into a West group and 40 populations into an East group (Fig. 2b and c). Principal coordinate analyses (PCoA) also suggested the populations should be clustered into two similar groups. The first two PCs explained 22.9% and 14.3% of the genetic variation, respectively (Fig. 2a). The genetic structure between groups was significant (Gp1, *p*-value = 0.024; Gp2, *p*-value = 0.0001). The Mantel test showed that isolation by distance was significant (*R*^2^ = 0.499, *P* = 0.001; Fig. S5).

**Table 1:** Sampling information, *n*SSR and cp.DNA genetic diversity, locality habitat suitability, stability obtained from SDMs for the *Quercus glauca* populations

Population ID	Longitude /Latitude	nSSR					cp.DNA				SDM		
		N	A_r_	H_e_	H_o_	C_A_	N	H_d_	π (×10^− 3^)	Haplotype	E_Pre_	E_LGM_	E_Stab_
**West group**													
1	98.48/24.75	-	-	-	-	-	8	0	0	H4(8)	0.72	0.67	0.95
2	100.12/23.57	14	3.45	0.56	0.49	0.43	10	0	0	H21(10)	0.74	0.40	0.66
3	100.32/23.66	13	3.04	0.51	0.42	0.23	11	0.33	0.28	H1(9)H4(2)	0.78	0.61	0.84
4	100.79/22.68	7	3.51	0.50	0.45	0.29	10	0.80	2.98	H4(4), H7(1), H8(1), H9(3), H10(1)	0.59	0.52	0.93
5	100.83/24.54	-	-	-	-	-	8	0.25	0.22	H1(1), H4(6), H22(1)	0.74	0.76	0.97
6	101.04/22.45	10	3.46	0.59	0.54	0.00	10	0.64	2.39	H28(5), H36(4), H44(1)	0.59	0.48	0.88
7	101.10/24.28	-	-	-	-	-	10	0.20	0.17	H1(1), H4(1), H22(8)	0.84	0.76	0.93
8	101.26/24.27	-	-	-	-	-	10	0	0	H45(10)	0.34	0.36	0.99
9	101.85/24.03	-	-	-	-	-	5	0	0	H4(5)	0.86	0.72	0.86
10	103.38/23.20	10	3.45	0.62	0.58	0.10	14	0.26	0.57	H1(2), H2(12)	0.86	0.50	0.63
11	104.57/24.46	9	3.50	0.61	0.66	0.78	10	0	0	H3(10)	0.87	0.57	0.69
12	104.85/22.98	10	3.69	0.62	0.60	0.10	10	0.20	0.17	H38(9), H43(1)	0.64	0.40	0.76
13	106.01/23.25	-	-	-	-	-	7	0	0	H24(7)	0.80	0.70	0.90
14	106.67/26.33	5	3.43	0.51	0.54	0.60	10	0	0	H24(10)	0.76	0.46	0.70
15	106.78/22.72	13	3.40	0.60	0.61	0.00	10	0.20	0.35	H56(9), H56(1)	0.88	0.74	0.86
16	107.94/25.29	11	4.26	0.71	0.63	0.18	10	0.78	1.93	H24(1), H31(4), H57(2)	0.97	0.81	0.84
17	109.17/29.67	6	2.99	0.43	0.38	0.50	8	0	0	H24(4), H30(4)	0.92	0.44	0.52
18	109.20/27.74	-	-	-	-	-	5	0	0	H36(5)	0.72	0.58	0.86
19	109.59/28.03	10	3.28	0.60	0.51	0.50	10	0.71	0.61	H36(1), H37(1), H1(3)	0.86	0.76	0.90
20	109.79/29.68	26	4.17	0.69	0.65	0.04	11	0.62	0.60	H24(6), H31(1), H36(4)	0.88	0.17	0.30
21	110.11/26.47	13	4.39	0.73	0.56	0.31	10	0.60	0.49	H23(3), H24(4), H30(1), H39(1), H40(1)	0.94	0.43	0.49
22	110.31/25.08	10	4.27	0.71	0.61	0.10	10	0	0	H30(1), H51(9)	0.96	0.91	0.96
23	110.47/24.39	6	3.26	0.59	0.61	0.00	-	-	-	-	0.98	0.90	0.93
24	110.48/29.13	10	4.00	0.63	0.59	0.00	10	0	0	H41(10)	0.82	0.69	0.87
25	110.5/24.78	10	3.67	0.63	0.72	0.00	10	0	0	H40(10)	0.96	0.90	0.94
26	110.75/26.28	8	5.97	0.54	0.54	0.50	-	-	-	-	0.94	0.71	0.77
**East group**													
27	112.48/23.11	-	-	-	-	-	8	0	0	H53(8)	0.88	0.73	0.85
28	112.87/24.81	-	-	-	-	-	12	0.30	0.13	H25(2), H30(10)	0.97	0.76	0.79
29	112.88/24.85	8	3.89	0.61	0.64	0.75	-	-	-	-	0.97	0.57	0.60
30	112.95/25.38	24	4.91	0.77	0.58	0.04	10	0.69	0.63	H14(5), H24(1), H30(2), H41(2)	0.93	0.80	0.87
31	113.75/25.04	5	3.57	0.52	0.49	1.00	8	0.25	0.11	H4(6), H5(1), H6(1)	0.94	0.85	0.91
32	113.99/24.03	10	2.88	0.52	0.53	1.00	10	0	0	H38(10)	0.98	0.77	0.79
33	114.20/26.52	10	3.74	0.57	0.57	0.80	10	0.20	0.09	H30(9), H52(1)	0.74	0.48	0.74
34	114.24/24.82	9	3.87	0.61	0.62	0.89	9	0	0	H24(9)	0.97	0.81	0.84
35	114.26/31.93	10	2.93	0.48	0.47	1.00	10	0.20	0.35	H1(1), H31(9)	0.58	0.12	0.54
36	114.28/22.65	-	-	-	-	-	9	0	0	H25(9)	0.97	0.71	0.74
37	115.21/25.21	10	3.56	0.61	0.53	1.00	-	-	-	-	0.97	0.77	0.80
38	115.45/25.02	9	3.93	0.63	0.53	0.89	-	-	-	-	0.97	0.75	0.78
39	115.46/29.34	-	-	-	-	-	8	0.25	0.32	H29(1), H42(7)	0.43	0.40	0.97
40	115.76/31.60	8	3.61	0.55	0.54	1.00	-	-	-	-	0.69	0.18	0.49
41	115.78/31.13	9	3.84	0.65	0.62	1.00	10	0	0	H31(10)	0.93	0.37	0.44
42	116.13/24.70	10	3.34	0.54	0.57	0.90	-	-	-	-	0.93	0.81	0.88
43	116.57/26.55	9	3.88	0.59	0.57	0.89	9	0.56	0.24	H4(5), H6(4)	0.93	0.54	0.61
44	116.72/25.36	10	3.76	0.60	0.55	0.90	11	0.69	0.60	H14(5), H24(2), H31(4)	0.96	0.76	0.80
45	117.36/27.65	10	3.64	0.61	0.54	1.00	10	0.20	0.09	H35(9), H54(1)	0.93	0.77	0.84
46	117.46/26.18	10	3.89	0.64	0.61	1.00	-	-	-	-	0.80	0.81	0.99
47	117.49/29.84	10	3.83	0.57	0.56	1.00	10	0.53	0.69	H25(6), H29(4)	0.79	0.22	0.43
48	117.53/25.99	17	4.04	0.70	0.69	0.00	10	0.64	1.02	H14(4), H29(4), H31(1), H33(1)	0.89	0.38	0.49
49	117.54/25.21	10	3.77	0.59	0.53	0.90	-	-	-	-	0.96	0.74	0.78
50	117.67/27.73	10	3.34	0.55	0.51	0.90	10	0.87	1.64	H25(2), H26(1), H27(2), H29(1), H34(2), H35(2)	0.73	0.43	0.70
51	117.75/27.68	10	3.77	0.57	0.57	0.90	10	0.60	0.75	H14(1), H25(6), H31(3)	0.84	0.54	0.70
52	117.91/29.13	10	4.13	0.64	0.64	1.00	10	0	0	H27(10)	0.87	0.55	0.69
53	118.53/25.75	10	3.97	0.67	0.69	0.90	-	-	-	-	0.97	0.55	0.58
54	118.71/26.28	10	3.38	0.54	0.51	1.00	-	-	-	-	0.94	0.44	0.50
55	118.82/25.54	9	3.86	0.64	0.58	1.00	-	-	-	-	0.96	0.49	0.53
56	118.82/27.23	10	3.70	0.59	0.43	0.90	-	-	-	-	0.94	0.67	0.74
57	119.03/28.09	10	3.93	0.58	0.60	1.00	10	0.78	0.75	H25(3), H31(4), H40(2)	0.89	0.90	0.99
58	119.26/28.44	-	-	-	-	-	10	0.47	0.61	H25(6), H46(1), H47(3)	0.96	0.67	0.71
59	119.37/26.06	10	3.13	0.54	0.49	1.00	10	0.20	0.17	H24(9), H32(1)	0.93	0.64	0.71
60	119.37/28.68	10	3.94	0.59	0.59	0.90	9	0	0	H29(9)	0.93	0.74	0.80
61	119.61/30.30	-	-	-	-	-	10	0	0	H49(10)	0.60	0.17	0.57
62	120.37/28.48	-	-	-	-	-	10	0.51	0.41	H25(2), H30(7), H50(1)	0.96	0.82	0.86
63	120.61/29.63	10	3.55	0.55	0.44	1.00	-	-	-	-	0.83	0.24	0.41
68	121.06/29.28	10	3.81	0.62	0.62	0.90	-	-	-	-	0.94	0.46	0.53
69	121.14/30.61	10	4.03	0.65	0.70	0.90	-	-	-	-	0.62	0.10	0.48
77	122.21/30.02	16	3.45	0.60	0.56	1.00	10	0	0	H25(10)	0.72	0.31	0.59
**Taiwan group**													
64	120.72/22.18	-	-	-	-	-	8	0	0	H19(8)	0.90	0.63	0.73
65	120.79/24.17	10	3.63	0.63	0.58	0.70	6	0.60	0.61	H1(4), H4(1), H6(1)	0.97	0.49	0.52
66	120.80/22.21	13	3.75	0.62	0.59	0.46	7	0.48	1.03	H14(2), H19(4), H2(1)	0.96	0.80	0.83
67	120.84/24.18	8	3.30	0.58	0.71	0.13	-	-	-	-	0.98	0.55	0.57
70	121.14/23.03	-	-	-	-	-	5	0.70	0.52	H4(1), H14(3), H15(1)	0.98	0.85	0.87
71	121.14/24.03	9	3.80	0.65	0.65	1.00	10	0.64	0.40	H6(5), H12(4), H13(1)	0.98	0.63	0.65
72	121.21/24.76	10	4.39	0.68	0.64	0.60	-	-	-	-	0.98	0.91	0.93
73	121.30/23.14	7	3.40	0.56	0.51	0.71	-	-	-	-	0.98	0.73	0.75
74	121.37/24.81	-	-	-	-	-	7	0	0	H11(7)	0.98	0.91	0.93
75	121.47/23.48	11	4.08	0.63	0.65	0.55	11	0.35	0.24	H1(5), H16(1), H17(1), H18(4)	0.98	0.92	0.94
76	121.68/24.99	8	4.16	0.65	0.79	0.50	-	-	-	-	0.98	0.84	0.86

*Notes* N: number of sampled individuals, *A*_r_: standardized allelic richness, *H*_e_: expected heterozygosity, *H*_o_: observed heterozygosity, *C*_A_: cluster A, *H*_d_: haplotype diversity, π: nucleotide diversity, *N*_Pre_: present habitat suitability, *N*_LGM_: LGM habitat suitability, *N*_Stab_: habitat stability since LGM, “-”: data missing

### Genetic diversity and differentiation based on cp.DNA markers

**Fig. 3:**
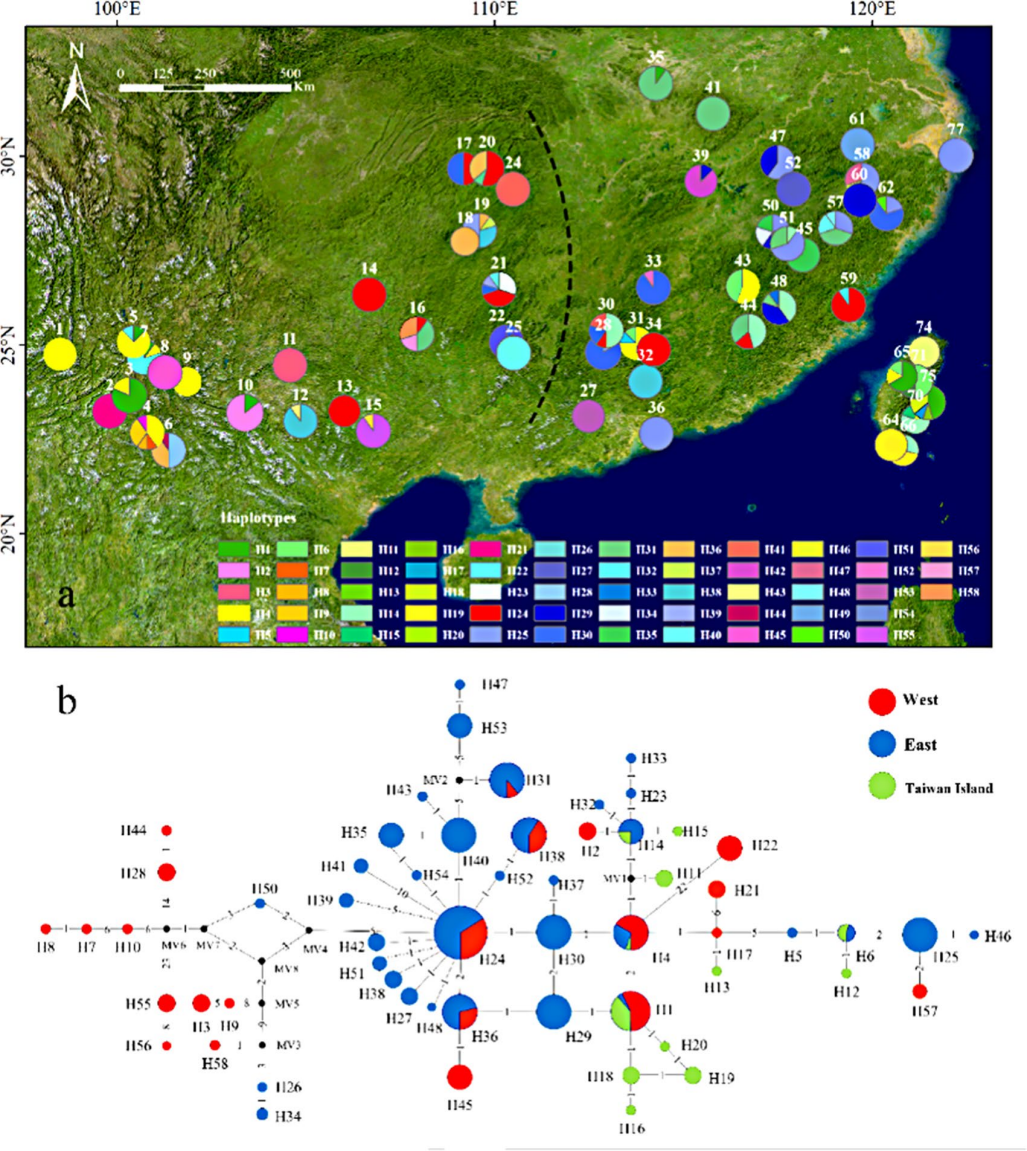
Distribution of the cp.DNA haplotypes detected in *Quercus glauca*. **a** geographic distribution of 58 cp.DNA haplotypes; **b** haplotypes of the cp.DNA network of *Q. glauca*. The haplotype network was colored according to the three regions delineated on the map (West, East, and Taiwan island). The size of points representing populations on maps and haplotypes in networks are proportional to the number of individuals. Numbers on the branches indicate the number of substitutions. Black spots indicate unsampled or extinct ancestral haplotypes

Three chloroplast loci were sequenced in 534 individuals from 57 populations (Table 1). The concatenated sequence was 2,437 bp total after alignment, and the lengths of the *psb*A-*trn*H, *trn*T*-trn*L, and *atp*I-*atp*H regions were 506 bp, 780 bp, and 1,151 bp, respectively. A total of 58 haplotypes with 40 variable sites were detected (Fig. 3a). Overall π and *H*_d_ of the populations were 1.22 × 10^− 3^ and 0.96, respectively. The highest *H*_d_ was observed in populations 50 (*H*_d_=0.87) and 4 (*H*_d_=0.80), and the highest π was found in populations 4 (π = 2.98 × 10^− 3^) and 6 (π = 2.39 × 10^− 3^; Table 1). Spatial genetic interpolation results showed higher *H*_d_ and π in the Nanling Mountains, Wuyi Mountains, and the southern region (Fig. S6). Overall diversity (*H*_T_) and diversity within populations (*H*_S_) based on cp.DNA were 0.859 and 0.286, respectively. The *N*_ST_ value (0.68) was only slightly higher than the *G*_ST_ value (0.67) and the difference was not significant (*p* > 0.05), suggesting the absence of a strong phylogeographic signal. Spatial distributions of haplotypes, including 18 shared and 40 private haplotypes, were arranged in a star-like structure (Fig. 3b). Haplotype 24 was the most widely distributed, found in 53 individuals from 10 populations. A total of 13 haplotypes were derived from haplotype 24. The second most frequent was haplotype 25, found in 46 individuals from 9 populations. Populations 50, 4, and 21 had the most haplotypes (*N* = 6, 5, 5, respectively), and 21 populations contained one haplotype.

**Fig. 4:**
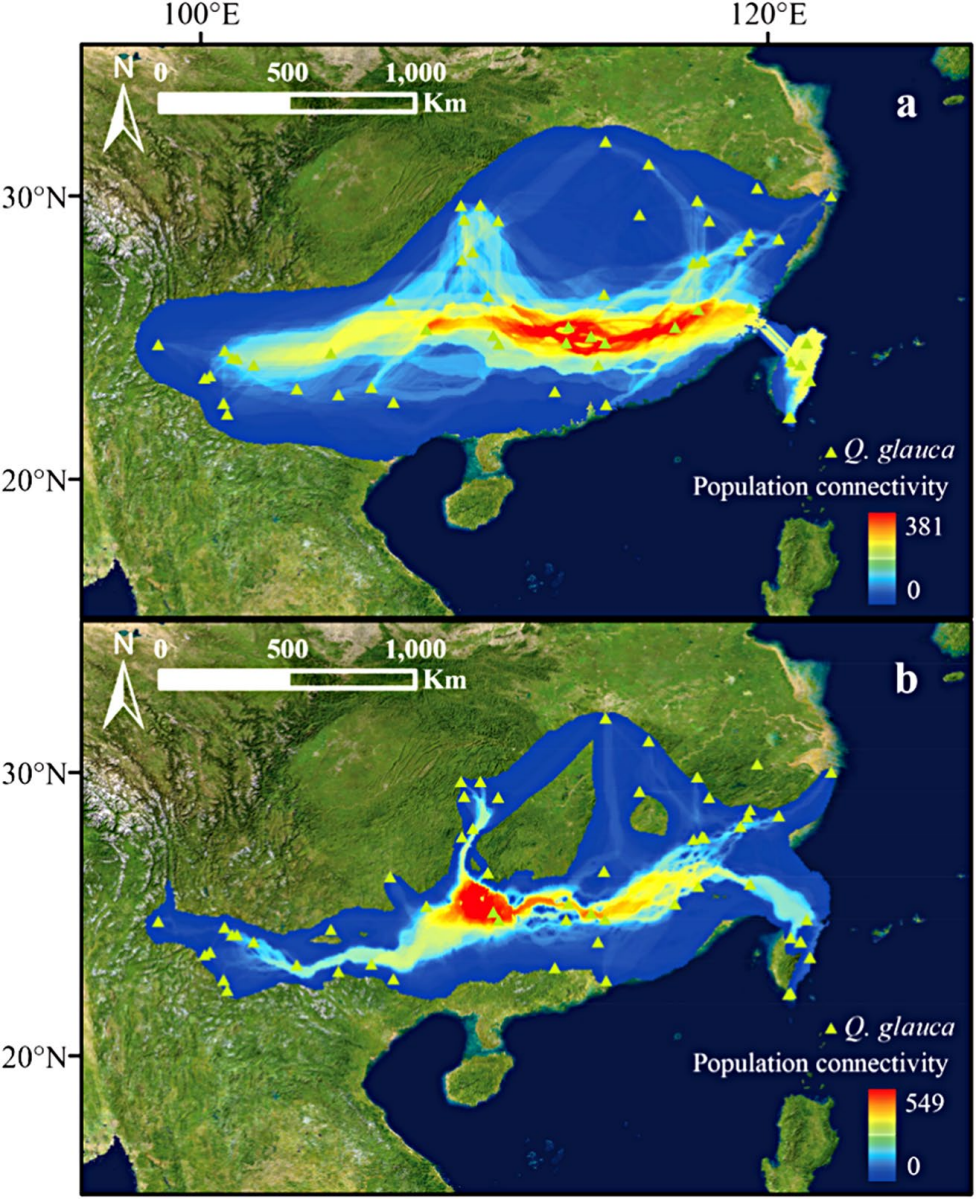
Potential dispersal corridors of the *Quercus glauca* during (**a**) the present and (**b**) the LGM period. Colors from blue to red represent the probability of species dispersal corridors from low to high

**Fig. 5:**
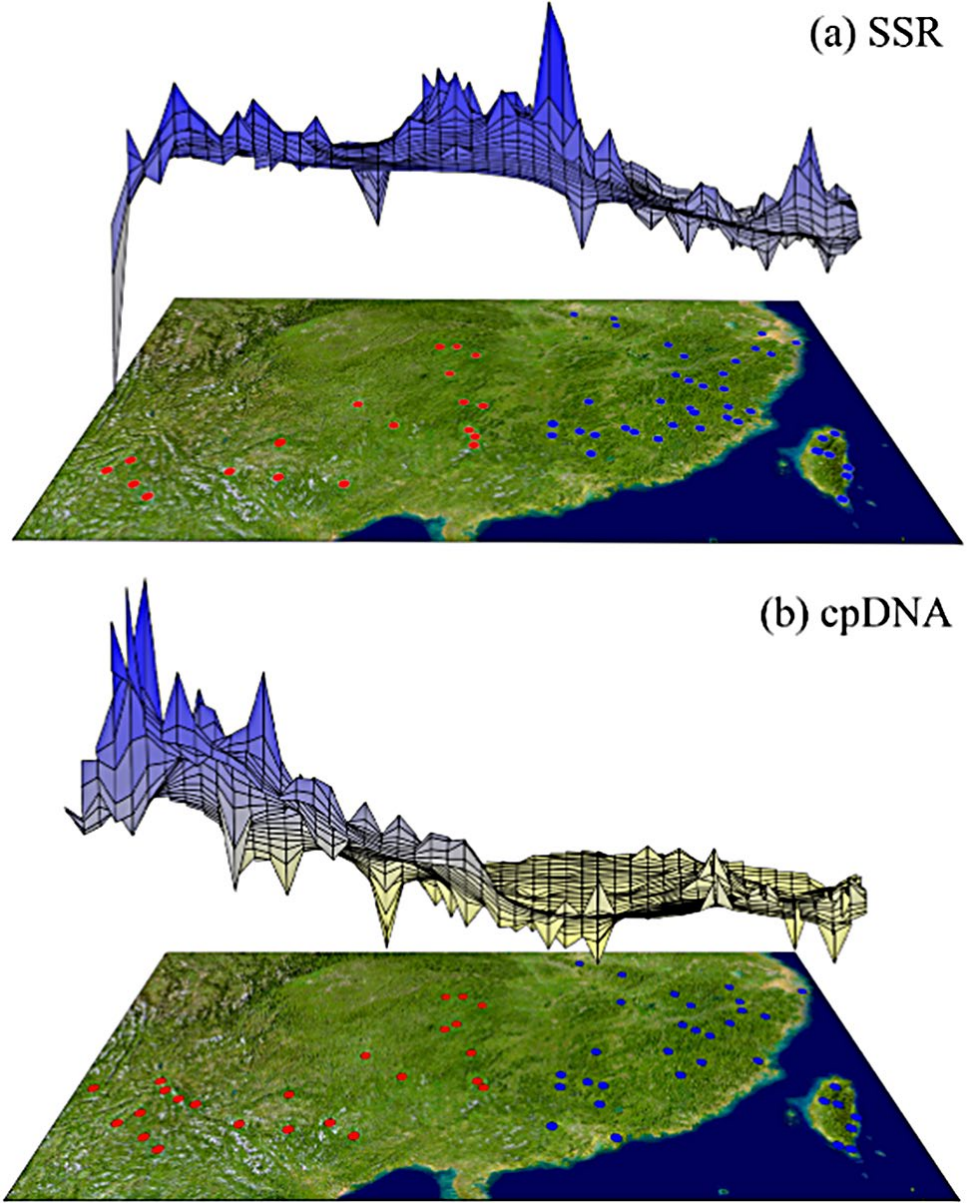
Genetic landscape shape analysis of *Quercus glauca* population based on (**a**) *n*SSR and (**b**) cp.DNA. The x-axis and y-axis represent the geographical location, and a Delaunay triangulation network is established between the sampled populations. Land surface height reflects the genetic distance between populations

Based on the species distribution model and the geographical distribution of haplotypes, the least-cost path method (LCP) method was used to calculate the possible migration and diffusion routes of *Q. glauca* from the LGM to the present period (Fig. 4). The results showed that *Q. glauca* migrated across the Yunnan Guizhou Plateau and the Nanling Mountains from the LGM to the present, as east-west mountains provide the main diffusion corridors. Genetic landscape shape analysis based on *n*SSR and cp.DNA data showed that genetic distances within the West population were higher than those within the East population. Notably, different patterns of population differentiation were detected between cp.DNA and *n*SSR markers (Fig. 5). For cp.DNA, genetic distance significantly declined from the West to the East and showed a peak in southwest China. The *n*SSR genetic distance peaked around the Nanling Mountains area, consistent with the higher genetic diversity indices observed in the regions.

### Correlation between genetic diversity and climate factors

GLM analysis indicated that the present period suitable habitat (*E*_Pre_) was significantly correlated with *A*_r_ and *H*_e_ when other factors were controlled (Table 2). Habitat stability (*E*_Stab_) was significantly correlated with *C*_a_, but it negatively correlated with *H*_e_. The model that best explained *C*_a_ included *E*_Stab_, longitude, and latitude, and the correlation with longitude was the strongest result. Longitude’s effect on genetic differentiation was greater than that of *E*_Stab_. For *H*_e_, *E*_Pre_ and *E*_Stab_ were included in the final model. Only *E*_Pre_ was significantly correlated with *A*_r_.

**Table 2:** Correlation between genetic diversity, habitat and geographical factors of *Q. glauca*

	A_r_				H_e_				C_a_			
	Est	SE^a^	t	p	Est	SE^a^	t	p	Est	SE^a^	t	p
***E***_**Pre**_	1.13	0.32	3.48	< 0.001***	0.09	0.03	3.23	< 0.01 **	-	-	-	-
***E***_**Stab**_	-	-	-	-	-0.10	0.05	-2.11	< 0.05 *	0.54	0.23	2.36	< 0.05 *
**Longitude**	-0.03	0.01	-1.88	0.06	-	-	-	-	0.03	0.01	4.76	< 0.001 ***
**Latitude**	0.04	0.03	1.41	0.16	-	-	-	-	0.04	0.02	2.43	< 0.05 *

Significance code ‘***’ 0.001, ‘**’ 0.01, ‘*’ 0.05; SE^a^: standard error

## Discussion

### Species spatial genetic patterns

Genetic diversity is essential for a species’ survival in the face of environmental changes and, as a result, for maintaining its role in ecosystem functions [40]. High genetic diversity (*H*_T_=0.859 and *A*_r_=2.88–5.97 in 620 individuals from 60 populations) of *Q. glauca* detected from *n*SSR data is similar to other oaks that are distributed in subtropical China, such as the *Q. franchetii* complex (*H*_T_=0.982 and *A*_r_=3.18–4.34 in 303 individuals from 33 populations) [41], *Q. delavayi* (*H*_T_=0.907 and *A*_r_=3.75–5.24 in 493 individuals from 33 populations) [42], and *Q. schottkyana* (*H*_T_=0.828 and *A*_r_=4.83–7.78 in 380 individuals from 29 populations) [11]. For cp.DNA, the number of haplotypes in this study was higher than in other oaks, such as *Q. aliena* with 33 haplotypes in 52 populations [43], *Q. fabri* with 21 haplotypes in 31 populations [44], *Q. acutissima* with 19 haplotypes in 30 populations [45]. Different genetic patterns in *n*SSR and cp.DNA might be related to sampling variance. Although the same cp.DNA loci were sequenced here, the number of haplotypes was higher in the previous study from 409 *Q. glauca* individuals of 42 populations [35]. The additional 125 samples in this study added 25 new haplotypes, mainly from the western region (10 haplotypes) and Taiwan Island (9 haplotypes). Most of the additional haplotypes were private. Long-term geographical or environmental isolation could drive in-situ diversification and maintain private haplotypes as species adapt to local environments [46–48].

No significant phylogeographic structure was evident based on cp.DNA (*N*_ST_>*G*_ST_, *p* > 0.05), a result consistent with other subtropical evergreen species such as *Q. variabilis* (*N*_ST_=0.855 > *G*_ST_=0.852, *p* > 0.05) [49], *Q. acutissima* (*N*_ST_=0.689 > *G*_ST_=0.630, *p* > 0.05) [45], and *Q. chenii* (*N*_ST_=0.888 > *G*_ST_=0.863, *p* > 0.05) [50]. In contrast, the genetic structure in *n*SSR data revealed a distinct pattern: 60 *Q. glauca* populations were split between two clusters representing the western and eastern portions of the sample area. The different genetic patterns detected in cp.DNA and *n*SSR makers are not unexpected. Because of their modes of inheritance, for instance, the markers have different dispersal patterns. Chloroplast DNA disperses through seeds and the nuclear genomes are transmitted via seeds and pollen. Oaks are prolific pollen producers and depend on wind for dispersal [51]. High levels of recent and ongoing gene flow should be evident in genetic patterns detected in nuclear markers, especially highly polymorphic SSRs [52]. To this latter point, more information may be available from *n*SSR than cp.DNA as the mutation rate in the former increases testable variation [53]. Meanwhile, the chloroplast genome is haploid with no recombination and has a smaller effective population size, which means it should be more sensitive to genetic drift and differentiation [54]. Therefore, differences in heredity between the nuclear and chloroplast genomes account for the different phylogeographic structure patterns we observed in the present study.

### Genetic variation and environmental heterogeneity

Species with long generation times and large effective population sizes, such as oaks, are likely delayed in their responses to environmental change [55]. Consequently, contemporary genetic patterns are often explained by past environmental conditions [56, 57]. In *Q. glauca*, however, habitat stability since the LGM (*E*_Stab_) was negatively correlated with genetic diversity (*H*_e_, *p* < 0.05), a surprising result considering the expectation that more climatically stable regions will retain higher genetic diversity [58, 59]. In addition to accumulating mutations over time, interspecies hybridization and admixture between populations can also increase genetic diversity [60]. Hybridization and introgression are common in oaks [32], and since East Asia is the main distribution range of the *Quercus* section *Cyclobalanopsis*, many closely related oaks have a sympatric distribution with *Q. glauca* [60, 61]. The distribution overlap of *Q. glauca* and closely related species affords the possibility of introgression. Range changes by *Q. glauca* since the LGM may have contributed to admixture among populations from different lineages. These factors could result in a negative correlation between genetic diversity and habitat stability. Similar results were also found in other oaks, such as *Q. kerrii* [62] and *Q. schottkyana* [11], in which the migration and mixing of genetically differentiated populations in unstable habitat areas led to increased genetic diversity.

Two distinct geography-related genetic lineages, West and East, were detected in *Q. glauca* populations from *n*SSR data. Genetic differentiation among populations in the western region is higher than in the eastern region. More complex topography in the west than in the east could provide higher stability for longer persistence and preservation of genetic variation, which may have facilitated the divergence of lineages through glacial/interglacial cycles [63–65]. Meanwhile, long-term environmental heterogeneity along altitudinal or latitudinal gradients also contributes to species genetic differentiation [62]. Distinct east-west phylogeographic differentiation has also been observed in other plants, such as *Dysosma versipellis*, *Cephalotaxus oliveri* and *Liriodendron chinense* [66–68]. Similar genetic patterns from different species indicated that topography and environmental heterogeneity could be the main drivers for intra-species differentiation in East Asia.

### Species distribution dynamics and dispersal route

Range shifts are a primary response by species to environmental change [69, 70]. The fossil record reflects numerous examples in both pollen and fossilized remains of species distribution shifts [71]. Combining species distribution models with spatial genetic patterns is useful for reconstructing distribution dynamics and dispersal routes [72, 73]. Although the eSDM inferred the suitable habitat of *Q. glauca* has shifted northeastward since the LGM, the distribution centroids analysis revealed that this species underwent significant expansion in the southwestern region. This suggests a pattern of northward expansion and southward retreat experienced by the species during the glacial/interglacial period [74]. The potential distribution of *Q. glauca* in the future under a broad but realistic climate scenario is predicted to shrink and move northeast. According to Ni [75], average temperature increases in China by 0.6–6.3℃ by the end of the 21st century will result in northward movements by forests and the formation of tropical rainforest climate in low latitudes. These predictions are supported by studies of other oaks, such as *Q*. *fabri* [76] and *Q*. *acutissima* [77].

The Nanling Mountains is a latitudinally arranged mountain range [78] that is commonly found to be a main route for species dispersal during glacial periods [79]. This is also the case for *Q. glauca*, which dispersed between southwest and southeast China between differing climate periods. Southwest China has been proposed as a potential glacial refugium and is regarded as the “cradle” of East Asian flora [80]. This region is also the biodiversity center of section *Cyclobanalopsis* [81], including through evidence from the fossil record such as with *Q. preglauca* (found in Sichuan, western China from the Pliocene and early Pleistocene, 4.5–2.3 Ma) [82] and *Q. preschottkyana* (found in Yunnan, southwest China from the late Miocene, about 11.5 Ma) [83]. Therefore, the direction of *Q. glauca* expansion from the LGM was from southwest to southeast China. Taiwan, meanwhile, is the largest subtropical mountainous island at the Tropic of Cancer in the monsoonal western Pacific region, and it emerged during the late Miocene and maintained a connection to the mainland at the latest in the Tertiary Pliocene [84–86]. The flora of Taiwan Island is most similar to the Southeast China [87]. The island now has 181 species of angiosperms in 20 families and 63 genera, of which 19 families, 62 genera, and 83 species are common to mainland China [88]. The Taiwan Strait (TS), which is ca. 130 km wide at its narrowest point and has a depth ranging between ca. 50–160 m, is the main migration route between Taiwan and southern China. The most extensive land bridges in the TS, as well as the East and South China Sea, would have formed during the LGM period when sea level was ca. 130 m lower [89]. For *Q. glauca*, the TS was the conduit for dispersal and gene flow between the island and mainland during the LGM. Moreover, phylogeographic studies on *Quercus championii* [90], *Dysosma versipellis-pleiantha* [91], *Juglans cathayensis* [92] have revealed high genetic similarity on both sides of the TS.

## Conclusions

This study sheds light on spatial genetic patterns and historical distribution dynamics of *Q. glauca*. The cp.DNA and *n*SSR data revealed that the *Q. glauca* has high genetic diversity and can divided into two distinct groups. Environmental heterogeneity contributed to the spatial genetic patterns observed. Interglacial warming promoted the spread of *Q. glauca* from western to eastern China. The Nanling Mountains may been both a dispersal corridor and glacial refugium, and the land bridges between mainland China and Taiwan islands during glacial periods may have contributed to the expansion of *Q. glauca* from the former to the latter. These results provide new insights into phylogeographic patterns and evolutionary histories of biotas in Southern China and adjacent islands, but also helpful for the management of forest biodiversity of East Asia subtropical EBLFs in the face of future climate change.

## Materials and methods

### Ensemble species distribution model

Distribution records of *Q. glauca* were obtained from field surveys, published documents, and records in the Chinese Virtual Herbarium (www.cvh.org.cn/). To reduce the effect of sampling bias on the prediction results, only one point was selected in each cell (size: 0.1°×0.1°). Nineteen bioclimatic variables with a 2.5’ resolution for the present (1970–2000s), LGM (Community Climate System Model version 4, CCSM4), and the future period (2081–2100s; Shared Socioeconomic Pathways, SSPs; The Beijing Climate Center Climate System Model, BCC-CSM2-MR) were downloaded from WorldClim (http://www.worldclim.org/) [93]. The present and future bioclimatic variables were version 2.1 [94], and the LGM period was version 1.4 [95]. Four common socioeconomic pathways (SSP126, SSP245, SSP370, and SSP585) were averaged for future climatic time frames to generate an integrated scenario [96, 97]. The climate variables at occurrence points in the present period were extracted, and we tested for correlations among them using the R package “*dismo*” [98]. High correlation variables (|*r*|>=0.8) were removed before further analysis.

We used an ensemble forecasting approach using the R package “*biomod2*” [99] to predict *Q. glauca* distribution with nine model algorithms (Table S2). Each model runs ten times with random seeds. For each model, the distribution data is randomly divided into two datasets: 75% is training data, and the remaining 25% is testing data. To build reliable species distribution models, 10,000 pseudo-absence coordinates were randomly generated [100, 101]. The presence and absence points were set with equal weights. The area under the receiver operating curve (AUC) and the true skill statistic (TSS) were used to evaluate the performance of the model. Models with higher TSS (> 0.6) and AUC values (> 0.8) were selected to construct an ensemble model. The weighted value of each model was proportional to the TSS value when constructing the ensemble model [102]. Predicted habitat was divided into categories of marginal (0.2–0.4), moderate (0.4–0.6), and highly suitable (> 0.6). To quantify the distribution change of *Q. glauca*, we also used 0.5 as the threshold value to convert the continuous suitable habitat from different periods to a presence/absence distribution [103, 104]. The distribution centroids of *Q. glauca* in the LGM, present, and future periods were calculated and compared using SDMToolbox [105].

### Population sampling and genotyping

A total of 781 individuals were sampled from 77 populations, which covered nearly the entire range of subtropical China (Fig. 1). Sample identification was performed by Xiao-Long Jiang and Min Deng, and voucher specimens of each individual were preserved in the Herbarium of Shanghai Chenshan Botanical Garden (CSH). Fresh and healthy mature leaves were collected and dried with silica gel to preserve them until DNA extraction could be performed. Genomic DNA was extracted using a modified cetyltrimethylammonium bromide (CTAB) protocol [106]. Seven *n*SSR loci were genotyped in 620 individuals from 60 populations (Table 1). Primer sequences and amplification conditions for each primer set were described in Table S3. For the cp.DNA, a total of 534 samples from 57 populations were sequenced (Table 1), including 157 individuals (18 populations) newly collected and 377 samples (39 populations) used from a previous study [35]. Three primer pairs, *psb*A-*trn*H [107], *trn*T-*trn*L [108], and *atp*I-*atp*H [109], were amplified and sequenced following previous studies (Table S3) [35]. Successfully amplified PCR products of cp.DNA and *n*SSR markers were sequenced or genotyped by the Shanghai Majorbio Bio-pharm Technology Co., Ltd (Shanghai, China). All cp.DNA sequences were assembled and manually assessed for quality in Sequencher v5.4.6 (Gene Codes Corp., Ann Arbor, MI, United States), then aligned with Bioedit v7.2.5 [110]. We determined *n*SSR genotypes using GeneMapper v4.1 [111].

### Population genetic analysis based on *n*SSR

Variation coefficients of *n*SSR data for each locus and population were calculated by GenAlEx v6.5 [112], including *H*_e_ (expected heterozygosity) and *A* (number of alleles) for loci, *H*_e_, *H*_o_ (observed heterozygosity) for each population. To avoid bias from unbalanced sample sizes, *A*_r_ (allelic richness) for each population was calculated by rarefaction (here, *N* = 10) using HP-Rare [113].

We used Bayesian clustering and PCoA to analyze the genetic structure of *Q. glauca*. For the former, we used STRUCTURE v2.3.4 [114] with 100,000 burn-in generations followed by 200,000 MCMC iterations. The number of clusters (*K*) varied from 1 to 10 with ten repetitions for each *K* value. The best fit *K* was determined by STRUCTURE HARVESTER [115] using both Δ*K* and Ln Pr(*X*|*K*) method [116, 117]. The ten repetitions with the optimum *K* value were aligned using CLUMPP v1.1.2 [118] based on a greedy algorithm. We performed the PCoA using GenAlEx v6.5 based on the genetic distances among populations. The first two principal coordinates (Gp1 and Gp2) at the population level were visualized in R. We used a Student’s t-test in the “*dplyr*” package [119] to test these for significance. To examine whether there was a significant correlation between genetic divergence and geographic distance (i.e., isolation-by-distance; IBD), we used a Mantel test in GenAlEx based on *n*SSR data, with 9,999 permutations. Genetic distance was estimated as *F*_ST_/(1-*F*_ST_) implemented on samples grouped by locality. Geographic distance was log transformed to account for dispersal in a two-dimensional habitat [120]. The correlation between geographical and genetic distance was plotted, and the correlation coefficient (*r*) and *R*^2^ were estimated using GenAlEx v6.5.

### cp.DNA analysis

Haplotype diversity (*H*_d_) and nucleotide diversity (π) of *Q. glauca* populations were calculated by ARLEQUIN v3.5 [121]. The median-joining network of haplotypes was constructed using NETWORK v10.2.8 [122]. Each indel and inversion was treated as a single mutation. The geographical distribution of the cp.DNA haplotypes was mapped with ArcGIS v10.8. Alleles in Space [123] was used to examine the distribution of genetic differentiation across species ranges according to cp.DNA and *n*SSR data. We used the Landscape Genetics GIS Toolbox [124] in ArcGIS v10.8 to create genetic landscape surfaces through interpolation based on genetic diversity (*H*_d,_ π, *H*_e,_ and *A*_r_). The software DnaSP v6.0 [125] was used to calculate *G*_ST_ and *N*_ST_. A significantly larger *N*_ST_ than *G*_ST_ implies the presence of a significant phylogeographic structure [126].

### Habitat analyses

Climatic variation and habitat complexity have well-defined roles in influencing species demography and occupancy, and both have been used to project species distribution dynamics under climate change [127]. To estimate the correlation of genetic diversity (*A*_r_, *H*_e_) and genetic structure (cluster A, *C*_a_) with climate and geographic (latitude, longitude) factors, a general linear model (GLM) analysis was performed in R. Four variables, *E*_Pre_, *E*_Stab_, latitude, and longitude, were used as explanatory covariates. The *E*_Stab_ was calculated using the formula *E*_Stab_=1−|*E*_Pre_−*E*_LGM_|, considering *E*_Pre_ and *E*_LGM_ as habitat suitability at the present and LGM periods, respectively. These values were obtained using the “Extract by Points” tool in ArcGIS v10.8 from *Q. glauc*a predicted distribution layers [11]. The initial model included all covariates, and the most suitable models were determined based on a backward elimination procedure.

Combined with our projections of suitable habitat and the spatial distribution of haplotypes, potential dispersal routes of the *Q. glauca* populations were inferred using the LCP function in the SDMToolbox [128]. To calculate least-cost corridors (LCCs), the LCP was weighted by resistance values with cutoffs for inclusion into high-, mid-, and low-classes set at 5%, 2%, and 1%, respectively. The weighted and categorized LCPs were then summed to create a LCCs dispersal network [129].

### Electronic supplementary material

Below is the link to the electronic supplementary material.

Supplementary Material 1

## Data Availability

The haplotype sequences used in this study were submitted to NCBI under accession numbers PP213780-PP213811. Meanwhile, the combined sequences of 58 haplotypes from three cpDNA sequences, nSSR data and code of SDM analysis submitted to FigShare ( 10.6084/m9.figshare.24798462.v2).
