# Supplementary material for: Ensemble species distribution modeling and multilocus phylogeography provide insight into the spatial genetic patterns and distribution dynamics of a keystone forest species, Quercus glauca
Source: BMC Plant Biol. 2024 Mar 4;24:168. doi: 10.1186/s12870-024-04830-1 (PMC10910841; doi:10.1186/s12870-024-04830-1)
Supplement: Supplementary file 1 — Supplementary Material 1 [file 12870_2024_4830_MOESM1_ESM.docx]

The following Supporting Information is available for this article:

Fig. S1 Nine dominant climatic factors affecting the potential suitable distribution of *Quercus glauca*.

Fig. S2 Prediction accuracy evaluation of different models.

Fig. S3 Prediction of potential suitable habitat of *Quercus glauca*. (a) the LGM period; (b) the present period (1970-2000s); (c) the future period (2081-2100s).

Fig. S4 Best fit *K* identified by both Δ*K* and Ln Pr(*X*|*K*). (a) Variation of ΔK as a function of K. (b) Logarithmic probability as a function of K.

Fig. S5 Fig. S5 Correlation between genetic distance [*F*_ST_/(1−*F*_ST_)] and geographical distance [Ln (km)] for 60 populations of *Quercus glauca*. The relationship was significant (Mantel test; *R*^2^=0.499, *P*=0.001).

Fig. S6 Spatial interpolation of genetic diversity of the *Quercus glauca* based on *n*SSR and *cp*DNA, (a) *H*_d_, (b) *π*, (c) *A*_r_, (c) *H*_e_. The color ranges from green to red, representing the genetic diversity values from low to high.

Table S1 The AUC and TSS values of the nine models for each running.

Table S2 Species distribution modelling algorithms used to build ensemble model in this study.

Table S3 *n*SSR and *cp*DNA primers used for *Quercus glauca*.


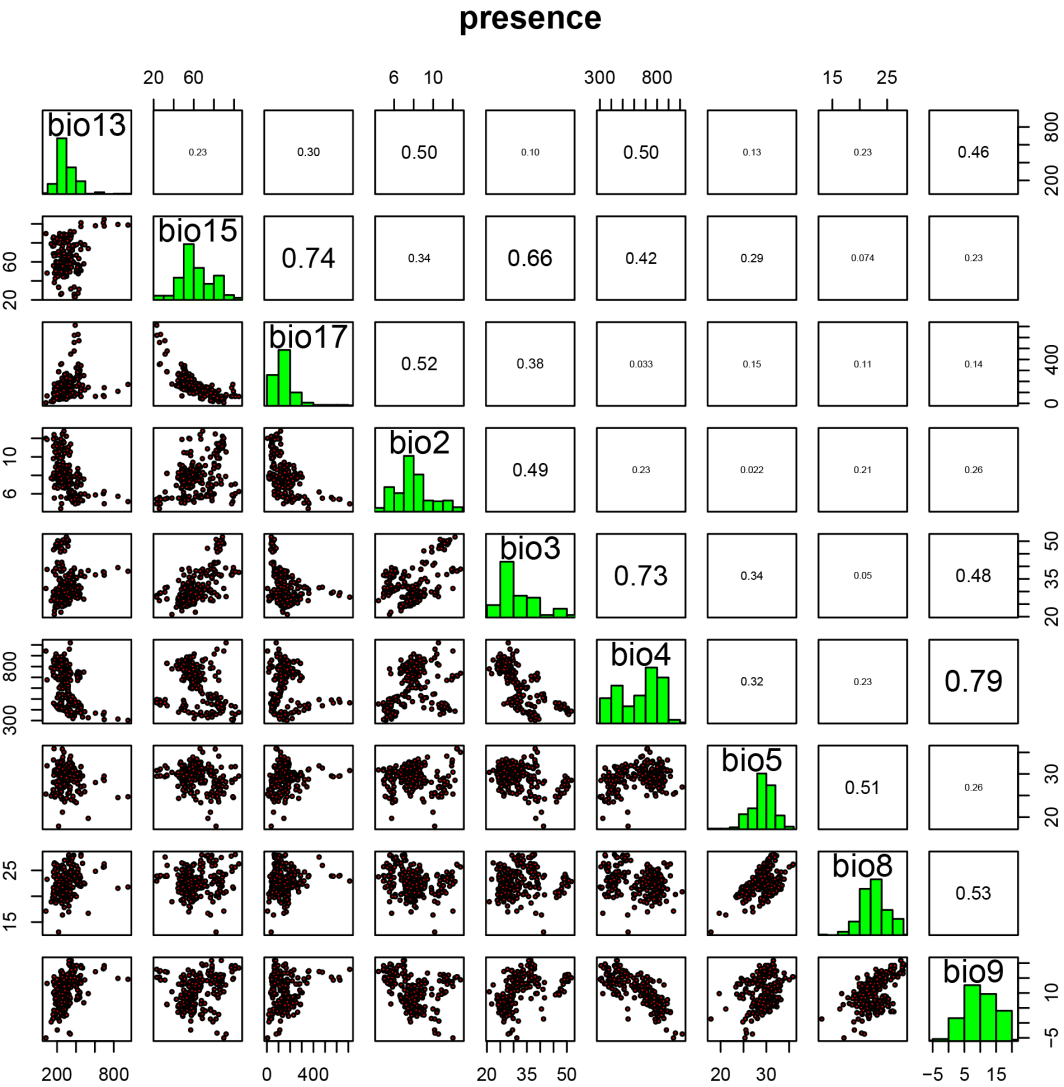


#### Fig. S1 Nine dominant climatic factors affecting the potential suitable distribution of *Q. glauca*.

####
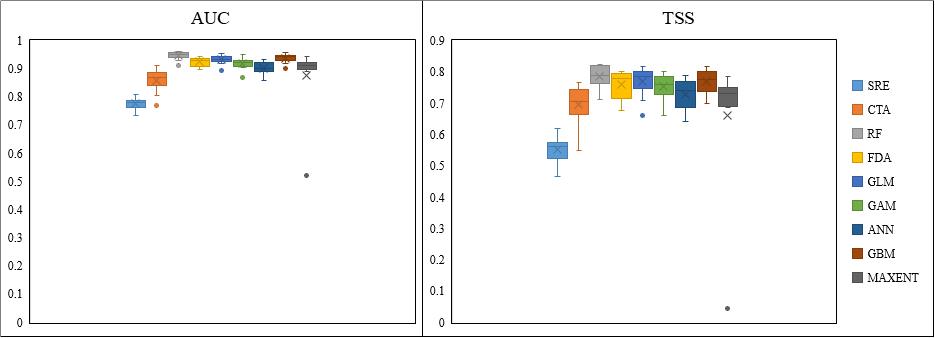
Fig. S2 Prediction accuracy evaluation of different models.

####
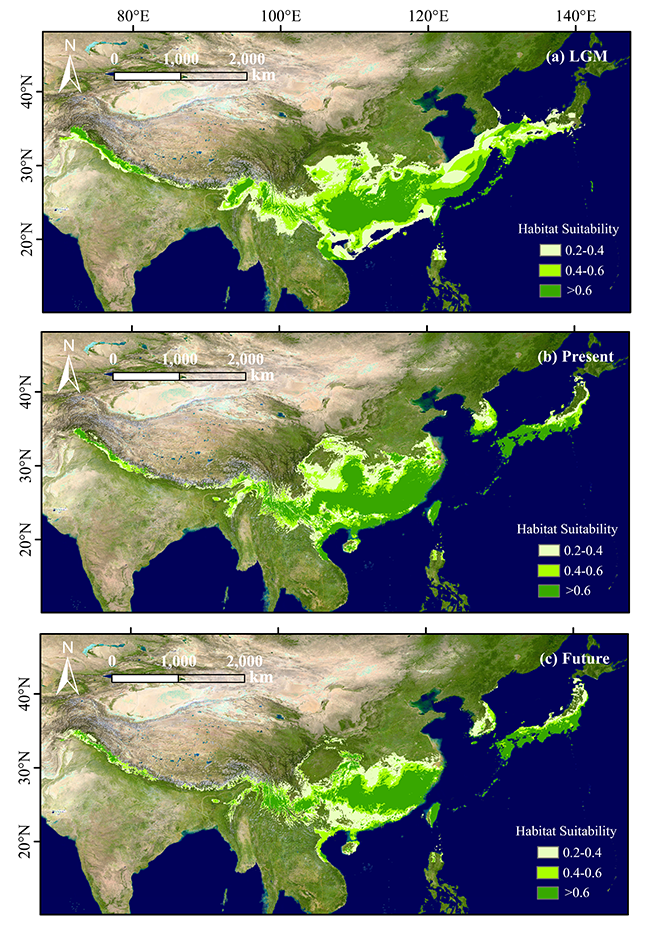
Fig. S3 Prediction of potential suitable habitat of *Q. glauca.* (a) the LGM period; (b) the present period (1970-2000s); (c) the future period (2081-2100s).


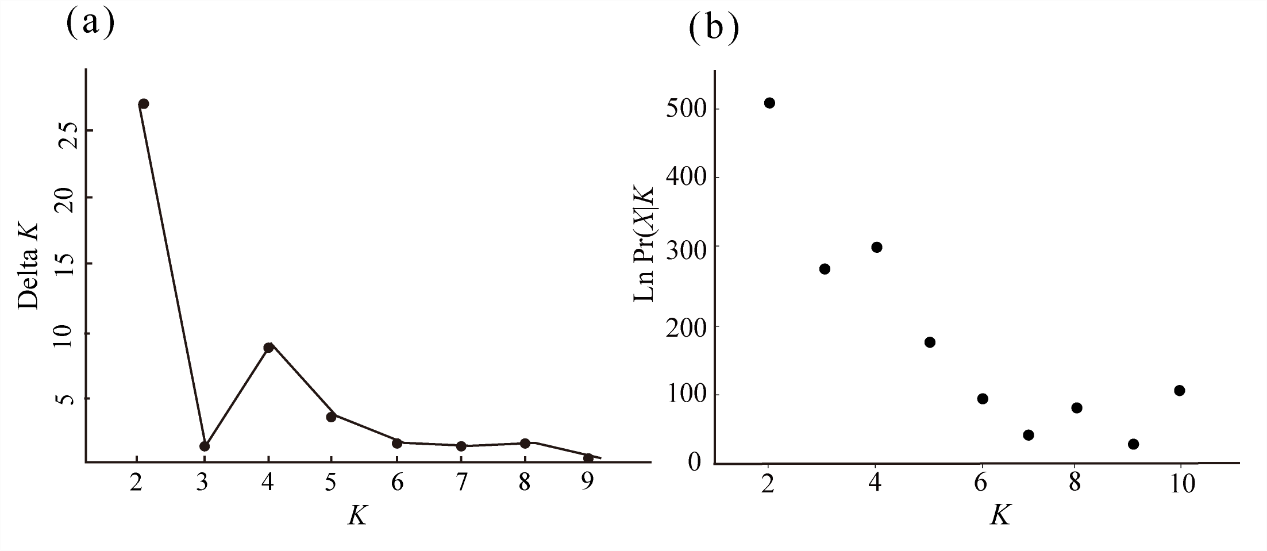


#### Fig. S4 Best fit *K* identified by both Δ*K* and Ln Pr(*X*|*K*). (a) Variation of ΔK as a function of K. (b) Logarithmic probability as a function of K.


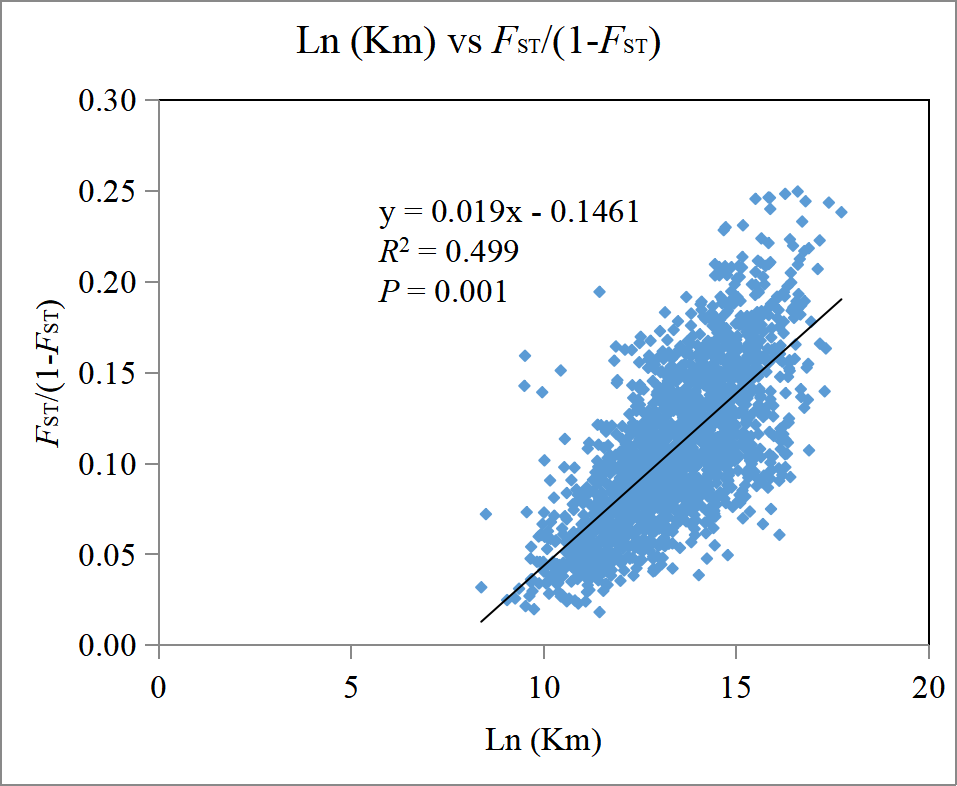


#### Fig. S5 Correlation between genetic distance [*F*_ST_/(1−*F*_ST_)] and geographical distance [Ln (km)] for 60 populations of *Quercus glauca*. The relationship was significant (Mantel test; *R*^2^=0.499, *P*=0.001).

####
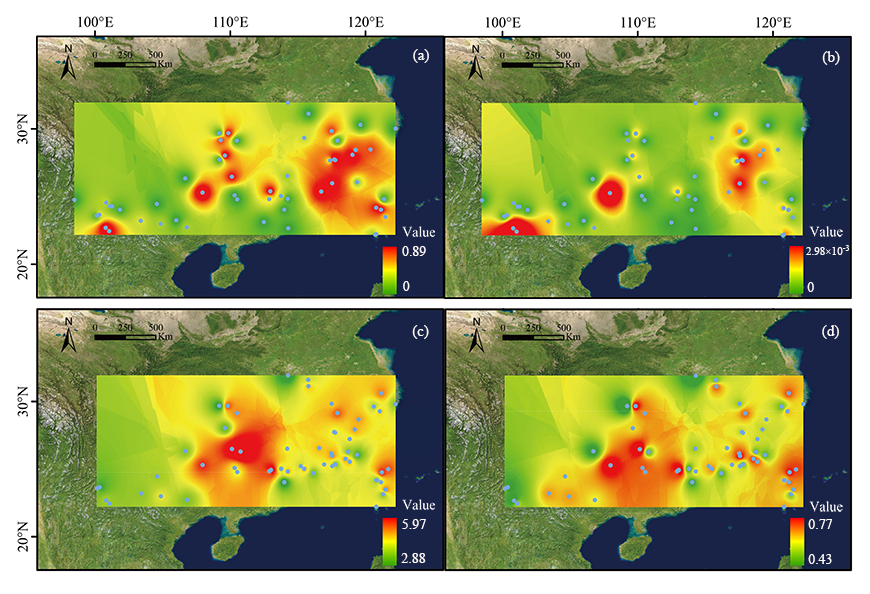
Fig. S6 Spatial interpolation of genetic diversity of the *Quercus glauca* populations based on *n*SSR and *cp*DNA, (a) *H*_d_, (b) *π*, (c) *A*_r_, (c) *H*_e_. The color ranges from green to red, representing the genetic diversity values from low to high.

Table S1 The AUC/TSS values of the nine models running ten times.

| Models | Run 1 | Run 2 | Run 3 | Run 4 | Run 5 | Run 6 | Run 7 | Run 8 | Run 9 | Run 10 |
| --- | --- | --- | --- | --- | --- | --- | --- | --- | --- | --- |
| SRE | **0.78/0.56** | **0.79/0.58** | **0.79/0.57** | **0.78/0.56** | **0.73/0.47** | **0.79/0.58** | **0.76/0.53** | **0.77/0.54** | **0.76/0.51** | 0.81/0.62 |
| CTA | 0.87/0.67 | 0.87/0.74 | 0.86/0.70 | 0.85/0.72 | **0.77/0.55** | 0.91/0.71 | 0.80/0.65 | 0.90/0.77 | 0.88/0.70 | 0.88/0.75 |
| RF | 0.95/0.77 | 0.96/0.82 | 0.96/0.77 | 0.94/0.80 | 0.91/0.71 | 0.96/0.82 | 0.94/0.78 | 0.96/0.82 | 0.93/0.75 | 0.96/0.81 |
| FDA | 0.91/0.73 | 0.93/0.78 | 0.93/0.77 | 0.94/0.80 | 0.90/0.68 | 0.94/0.79 | 0.93/0.79 | 0.93/0.78 | 0.9/0.680 | 0.95/0.80 |
| GLM | 0.93/0.78 | 0.93/0.80 | 0.94/0.76 | 0.93/0.76 | 0.89/0.66 | 0.95/0.79 | 0.93/0.81 | 0.94/0.80 | 0.92/0.71 | 0.95/0.82 |
| GAM | 0.93/0.80 | 0.91/0.77 | 0.92/0.73 | 0.93/0.78 | 0.87/0.66 | 0.95/0.76 | 0.91/0.75 | 0.93/0.80 | 0.90/0.72 | 0.93/0.77 |
| ANN | 0.90/0.73 | 0.90/0.74 | 0.89/0.64 | 0.93/0.79 | 0.86/0.70 | 0.93/0.78 | 0.90/0.76 | 0.92/0.75 | 0.87/0.65 | 0.91/0.77 |
| GBM | 0.93/0.75 | 0.94/0.80 | 0.95/0.77 | 0.94/0.78 | 0.90/0.70 | 0.96/0.82 | 0.93/0.77 | 0.95/0.79 | 0.92/0.72 | 0.95/0.82 |
| MAXENT | 0.92/0.73 | 0.94/0.79 | 0.91/0.70 | 0.92/0.74 | 0.89/0.69 | 0.94/0.75 | 0.91/0.73 | **0.52/0.05** | 0.90/0.69 | 0.91/0.75 |

Note: Bold characters indicate that models with lower evaluation were excluded.

Table S2 Species distribution modelling algorithms used to build ensemble model in this study.

| Class of model | | Method | | | | Description of sub-model as used in Biomod2 | | | | | | | | | |
| --- | --- | --- | --- | --- | --- | --- | --- | --- | --- | --- | --- | --- | --- | --- | --- |
| Artificial Neural Networks | | ANN | | | | A single-hidden-layer neural network that uses a five-fold internal cross-validation to choose the best number of units in the hidden layer and weight decay. These two parameters control model complexity. | | | | | | | | | |
| Classification Tree Analysis | | CTA | | | | A decision tree model fitted with default settings in the underlying rpart package. Under biomod2 defaults it fits complex trees with many nodes. A five-fold internal cross-validation is used to choose the best model. | | | | | | | | | |
| Flexible discriminant analysis | | FDA | | | | This method first fits a MARS model (fitted through mda package) then performs dimensionality reduction before attempting classification. | | | | | | | | | |
| Generalized Additive Model | | GAM | | | | A regression model that fits smoothed additive response curves through the mgcv package, allowing no interactions between covariates. | | | | | | | | | |
| Generalized boosted regression models | | GBM | | | | A machine-learning method that ensembles regression trees through gradient boosting. A maximum of 2500 relatively deep trees are fitted, and best iteration of trees is selected using an internal three-fold cross-validation. | | | | | | | | | |
| Generalized linear models | | GLM | | | | A regression model that fits quadratic response curves with no interactions between covariates, with stepwise backward selection using Akaike's information criterion. | | | | | | | | | |
| Maximum entropy | | MAXENT | | | | It is a machine-learning method that estimates the species distribution probability by assessing the maximum entropy distribution, so that the most spread-out, or closest to uniform. | | | | | | | | | |
| Random forests | | RF | | | | A machine-learning method that ensembles predictions from 500 classification trees, fitted on randomly selected subsets of all training data. Individual trees are controlled to have at least five data points in their terminal nodes, but are otherwise allowed to grow as many nodes as possible. | | | | | | | | | |
| Surface Range Envelope | | SRE | | | | Modeling methods for determining the extent of species by comparing and identifying extremes in species distribution data and environmental variable data. | | | | | | | | | |
| Table S3 nSSR and cpDNA primers used for Quercus glauca. | | | | | | | | | | | | | | |  |
|  |  | |  | PCR reactions system (unit: μl) | | | | | | | |  |  |  |  |
| Marker | Ta (°C) | | Total | Buf | Mg2+ | | dNTP | Pri+ | Pri- | Taq | DNA | *A* | He | Primer origin |  |
| nSSR |  | |  |  |  | |  |  |  |  |  |  |  |  |  |
| QrZAG7 | 59 | | 20 | 2 | 1 | | 0.3 | 0.5 | 0.5 | 0.2 | 1 | 19 | 0.57 | Kampfer et al. 1998 |  |
| QrZAG96 | 55 | | 20 | 2 | 1 | | 0.3 | 0.5 | 0.5 | 0.2 | 1 | 16 | 0.50 | Kampfer et al., 1998 |  |
| QpZAG36 | 50 | | 20 | 2 | 1 | | 0.3 | 0.5 | 0.5 | 0.2 | 1 | 15 | 0.66 | Steinkellner et al., 1997 |  |
| comp17611 | 59 | | 20 | 2 | 1 | | 0.3 | 0.5 | 0.5 | 0.2 | 1 | 9 | 0.61 | An et al., 2016 |  |
| comp19373 | 60 | | 20 | 2 | 1 | | 0.3 | 0.5 | 0.5 | 0.2 | 1 | 13 | 0.47 | An et al., 2016 |  |
| comp21430 | 59 | | 20 | 2 | 1 | | 0.3 | 0.5 | 0.5 | 0.2 | 1 | 9 | 0.47 | An et al., 2016 |  |
| comp31416 | 60 | | 20 | 2 | 1 | | 0.3 | 0.5 | 0.5 | 0.2 | 1 | 9 | 0.53 | An et al., 2016 |  |
| cpDNA |  | |  |  |  | |  |  |  |  |  |  |  |  |  |
| psbA-trnH | 55 | | 20 | 2 | 1 | | 0.3 | 0.5 | 0.5 | 0.2 | 1 | - | - | Shaw et al., 2005 |  |
| *trnT-trnL* | 55 | | 20 | 2 | 1 | | 0.3 | 0.5 | 0.5 | 0.2 | 1 | - | - | Taberlet et al., 1991 |  |
| atpI-atpH | 52 | | 20 | 2 | 1 | | 0.3 | 0.5 | 0.5 | 0.2 | 1 | - | - | Grivet et al., 2001 |  |

Notes: Ta, annealing temperature; Total, a total volume 20 μl of PCR mixtures; Buf, 10X PCR buffer; Mg2+, 10 mM MgCl_2_; dNTP, 2.5 mM dNTP each; Pri+, 10 μM forward primer; Pri-, 10 μM reversed primer; Taq, 5U/μl TaqDNA polymerase; DNA, 10ng/μl genomic DNA; *A*, number of alleles; *H*_e_, expected heterozygosity.

An M, Deng M, Zheng SS, Song YG. De novo transcriptome assembly and development of SSR markers of oaks *Quercus austrocochinchinensis* and *Q. kerri*i (Fagaceae). Tree Genet Genomes. 2016;12:103.

Kampfer S, Lexer C, Glössl J, Steinkellner H. Characterization of (GA) n microsatellite loci from *Quercus robur*. Hereditas. 1998;129:183-6.

Steinkellner H, Fluch S, Turetschek E, Lexer C, Streiff R, Kremer A, et al. Identification and characterization of (GA/CT) n-microsatellite loci from Quercus petraea. Plant Mol Biol. 1997;33:1093-96.

Grivet D, Heinze B, Vendramin GG, Petit RJ. Genome walking with consensus primers: application to the large single copy region of chloroplast DNA. Mol Ecol Notes. 2001;1:345-9.

Shaw J, Lickey EB, Beck JT, Farmer SB, Liu WS, Miller J, et al. The tortoise and the hare II: relative utility of 21 noncoding chloroplast DNA sequences for phylogenetic analysis. Am J Bot. 2005;92:142-66.

Taberlet P, Gielly L, Pautou G, Bouvet J. Universal primers for amplification of three non-coding regions of chloroplast DNA. Plant Mol Biol.1991;17:1105-09.
